# Enhancing enzymatic saccharification of sugarcane bagasse by combinatorial pretreatment and Tween 80

**DOI:** 10.1186/s13068-018-1313-7

**Published:** 2018-11-09

**Authors:** Hongdan Zhang, Weiqi Wei, Jiajie Zhang, Shihang Huang, Jun Xie

**Affiliations:** 1 College of Forestry and Landscape Architecture, Key Laboratory of Energy Plants Resource and Utilization, Ministry of Agriculture, South China Agricultural University, Guangzhou, 510642 People’s Republic of China; 2 State Key Laboratory of Pulp and Paper Engineering, South China University of Technology, Guangzhou, 510640 People’s Republic of China; 3 College of Light Industry and Food Engineering, Nanjing Forestry University, Nanjing, 210037 People’s Republic of China

**Keywords:** Sugarcane bagasse, Combinatorial pretreatment, Glucose, Tween 80

## Abstract

**Background:**

The recalcitrant structure of lignocellulosic biomass made it challenging for their bioconversion into biofuels and biochemicals. Pretreatment was required to deconstruct the intact structure by the removal of hemicellulose/lignin, improving the cellulose accessibility of enzyme. Combinatorial pretreatments with liquid hot water/H_2_SO_4_ and ethanol/NaOH of sugarcane bagasse were developed to improve enzymatic hydrolysis under mild conditions.

**Results:**

After one-step 60% ethanol containing 0.5% NaOH pretreatment with solid to liquid ratio of 1/10, the glucose yield after hydrolysis for 72 h with enzyme dosage of 20 FPU/g substrate was enhanced by 41% and 205% compared to that of NaOH or 60% ethanol pretreated solids, respectively. This improvement was correlated with the removal of hemicellulose and lignin. However, using combinatorial pretreatments with 1% H_2_SO_4_ followed by 60% ethanol containing 0.5% NaOH, the highest glucose yield with Tween 80 reached 76%, representing 84.5% of theoretical glucose in pretreated substrate. While retaining similar glucose yield, the addition of Tween 80 capacitated either a reduction of enzyme loading by 50% or shortening hydrolysis time to 24 h. However, the enhancement with the addition of Tween 80 decreased as hydrolysis time was extended.

**Conclusions:**

This study demonstrated that a combinatorial pretreatment with 1% H_2_SO_4_ followed by 60% ethanol containing 0.5% NaOH had significant effects on improving the enzymatic hydrolysis of sugarcane bagasse. The addition of Tween 80 enabled reducing the enzyme loading or shortening the hydrolysis time. This study provided an economically feasible and mild process for the generation of glucose, which will be subsequently converted to bioethanol and biochemicals.

**Electronic supplementary material:**

The online version of this article (10.1186/s13068-018-1313-7) contains supplementary material, which is available to authorized users.

## Background

Considering the energy challenges and environmental problems, it is imperative to explore sustainable energy derived from lignocellulosic biomass. Due to the abundant content of carbohydrates and lignin, they can be converted to value-added fuels, chemicals, and materials by biorefinery processes [1]. Among them, bioethanol production from lignocellulosic biomass has occupied a lead position as a viable option to petroleum fuels to relieve energy crisis and environmental problems. However, the matrix structure of lignocellulosic biomass prevented the enzymatic saccharification and subsequently fermentation to bioethanol [2]. Therefore, pretreatment is required to deconstruct the intact structure by removal of hemicellulose/lignin and improve the enzyme accessibility to cellulose [3, 4].

To date, various pretreatments have been developed, including liquid hot water pretreatment, dilute acid pretreatment, alkali-based pretreatment, ethanol pretreatment, steam explosion pretreatment, and ionic liquid pretreatment [3–7]. For example, liquid hot water pretreatment or dilute acid pretreatment could improve the enzymatic saccharification by dissolving hemicellulose. However, they could cause irreversible hemicellulose degradation and formation of inhibitors (such as formic acid, acetic acid, HMF, and furfural) [5]. For alkali-based, steam explosion, or organosolv pretreatment, though it did not cause corrosion, sugar degradation, and the formation of inhibitors, the simultaneous decomposition of hemicellulose and lignin made it difficult for the biorefining of all components. Considering these advantages and disadvantages, it is impossible to achieve integrated utilization of hemicellulose and lignin by one-step pretreatment. Hence, competitive two-stage pretreatment was proposed to degrade hemicellulose/lignin in separate two stages and recover their products, respectively.

Kim et al. investigated a two-stage fractionation processing using acetic acid at 170–190 °C for 10–20 min in the first step, and ammonium hydroxide at 140–220 °C for 5–25 min in the second step, which improved the enzymatic digestibility to 72.9% [8]. An integrated pretreatment of sweet sorghum stems with liquid hot water and NaOH yielded enzymatic saccharification of 77.5%, which was much higher than that obtained from individual pretreated substrates [2]. So far, most of these two-step pretreatment reports focused on determining how these two-step pretreatments were superior to individual one-step pretreatment on cellulose enzymatic digestibility, paid less attention to how one-step pretreatment affected the second-step pretreatment, and how to further improve the enzymatic hydrolysis of substrates after two-step pretreatment.

It was reported that the addition of surfactants, polymers or non-catalytic proteins to pretreated solids could improve their enzymatic digestibility by improving the activity and stability of cellulase, fortifying positive interactions between substrate and enzyme, or reducing unproductive enzyme binding [9–11]. The addition of PEG 8000 during hydrolysis led to the lignocellulose theoretical conversion of 67% after 24 h with a half reduction of enzyme loading [12]. Rocha-Martin et al. found that the addition of PEG 4000 increased the glucose yield and reduced the liquefaction time, ascribing to the increment activity of beta-glucosidase and endoglucanase by 20% and 60%, respectively [13]. Tweens were proposed to lubricate the access of cellulase to cellulose and subsequently combined with the free chemical groups released from lignin to prevent the adsorption of cellulase to lignin, and to provide more cellulase for cellulose [14]. Though the positive influence of additives on the enzymatic saccharification had been reported in previous research [12–14], systematic analyses accounting for the effect of additives on one-step and two-step pretreated substrates have been scarce.

Therefore, in this study, a two-stage pretreatment was proposed to hydrolyze hemicellulose using liquid hot water or 1% H_2_SO_4_ pretreatment at 120 °C for 30 min in the first step and then to degrade lignin using alkali (0.5% NaOH) or 60% ethanol or the combination of them at 120 °C for 60 min in the second step, thus improving the enzymatic saccharification of sugarcane bagasse. Then, the one-step and two-step pretreated substrates were characterized by chemical constituent analysis, X-ray diffraction (XRD), scanning electron microscopy (SEM), and thermogravimetric (TG) analysis. Simultaneously, the dissociation mechanisms of hemicellulose/lignin, and their structural features were thoroughly investigated to explore how they affected the enzymatic hydrolysis. Furthermore, the influence of Tween 80 on the enzymatic saccharification of one-step and two-step pretreated substrates was also determined.

## Results and discussion

### The chemical composition of pretreated solids and pretreatment liquors

The native sugarcane bagasse was found to contain 41.2 g of glucan, 20.2 g of xylan, and 22 g of AIL based on 100 g raw material. After various pretreatments, the chemical compositions in the residues were tested and found to be greatly altered, and the data were summarized and are depicted in Table 1. For pretreatment with 0.5% NaOH (Case 1), about 18.8% of xylan and 9.3% of AIL were degraded during the pretreatment process, contributing to the weight loss of 12.1%. When 60% ethanol pretreatment was conducted, the solid recovery reached 95.3%, due to the high recovery of xylan and AIL, suggesting that the low reaction temperature (120 °C) was not enough for the degradation of hemicellulose and lignin during the ethanol pretreatment process [15]. However, the 60% ethanol pretreatment containing 0.5% NaOH resulted in the enhancement of xylan removal (22.6%) and delignification (17.9%), leading to solid recovery of 82.8%. This phenomenon indicated that the addition of NaOH in ethanol pretreatment could dissolve lignin and hemicellulose effectively, increasing the enzyme accessibility to cellulose [16]. When liquid hot water pretreatment with combined severity factor of − 4.89 was employed, about 96.7% of solid was recovered, and only slight xylan and AIL were degraded, suggesting that the acidity provided by the hydronium during liquid hot water pretreatment was not enough for the hydrolysis of glycosidic linkages in hemicellulose at the mild conditions [17]. When the substrate pretreated with liquid hot water was used for the second-step pretreatment, the xylan removal was lower than that of native material as substrate; however, a significant enhancement in delignification was observed. This phenomenon showed that the removed lignin resurfaced on the fibers caused by liquid hot water pretreatment facilitated the delignification and impeded the hemicellulose removal [18]. The pretreatment with liquid hot water at 170–180 °C (close to the glass transition temperature) also resulted in the migration of lignin from the middle lamella and cell wall to the fiber surface [19]. However, the delignification after liquid hot water at higher temperatures was difficult due to lignin modified by condensation reactions, which was not consistent with our result, because there was no lignin modification in our research at 120 °C. For dilute acid (H_2_SO_4_) pretreatment with combined severity factor of 0.95, the xylan and AIL removal reached 75.5% and 1.3%, respectively, indicating that dilute acid pretreatment solubilized much more xylan (hemicellulose) but slight lignin, due to the higher acidity of H_2_SO_4_ [18]. Furthermore, after second pretreatment with 60% ethanol, or 0.5% NaOH, or the combination of them, the contents of xylan and AIL were significantly reduced, especially after the ethanol pretreatment enhanced by NaOH (Case 11). As shown in Table 1, the solid recovery decreased gradually after two-step pretreatment, due to the large removal of hemicellulose and lignin, which resulted in the aggregation of glucan contents in pretreated solids. More than 90% of glucan was found in pretreated substrates. Due to the large removal of hemicellulose and lignin in combinatorial treatments, more cellulose on the surface of fibers increased the enzyme accessibility. Also, the decreased lignin in the pretreated substrate would also reduce the unproductive binding of enzyme and increase the subsequent enzymatic hydrolysis. It was also reported by Acharjee et al. that the alteration of lignin hydrophobicity and the H-bond during ClO_2_ pretreatment induced association between the enzyme and lignin, leading to the unproductive binding of enzyme with lignin, consequently increasing the digestibility [4].

**Table 1:** Chemical composition of sugarcane bagasse before and after pretreatment under different conditions

Case	Solid recovery	Glucan (%)		Xylan (%)		AIL (%)	
		Content	Recovery	Content	Removal	Content	Removal
Material	100	41.2	–	20.2	–	22	–
Case 1	87.9	45.5	97.2	18.7	18.8	22.7	9.3
Case 2	95.3	42.2	97.5	18.3	13.4	22.5	2.5
Case 3	82.8	48.0	96.5	18.9	22.6	21.8	17.9
Case 4	96.7	42.0	98.5	20.1	3.8	22.4	1.5
Case 5	72.4	55.8	98.1	6.8	75.5	30.0	1.3
Case 6	83.9	46.8	95.3	22.1	8.1	16.3	37.8
Case 7	94.0	42.1	96.1	20.4	5.2	20.4	12.7
Case 8	79.6	49.0	94.6	20.3	19.9	10.9	60.5
Case 9	59.0	67.4	96.4	5.3	84.5	22.7	39.1
Case 10	67.7	59.4	97.7	5.8	80.6	25.0	23.1
Case 11	55.1	67.2	89.9	5.3	85.6	17.9	55.3

As stated in Table 1, a large amount of hemicellulose was removed during the pretreatment; hence, it is necessary to determine the degradation products of it. The results are depicted in Additional file 1: Table S1. As shown, the amount of xylose was higher than that of glucose, attributed to the major content of xylan in hemicellulose, and/or the strong cellulose, which was much more stable than xylan due to the crystalline structure and high degree of polymerization [18]. The highest xylose yield was obtained after 1% H_2_SO_4_ pretreatment, reached 3.14 g/100 g raw material (including 1.49 g monomer xylose and 1.65 g oligomer xylose), representing 18.2% of removed xylan. This relatively low recovery of xylan indicated that the majority of removed hemicellulose was just dissolved from the biomass, and the pretreatment condition was not enough for the adequate degradation of xylan to oligomer or monomer xylose. The absent furfural/HMF in the pretreatment liquor also verified the fact that the weak pretreatment conditions were inadequate for the dehydration of xylose/glucose to furfural/HMF. For other degradation components in hemicellulose, the recovery of arabinose and galactose in the pretreatment liquor was higher than that of xylose, suggesting that the arabinan and galactan in hemicellulose were easier to degrade than xylan, which was consistent with previous report that the removal of hemicellulose was ascribed to the cleavage of acetyl and arabinosyl groups [20]. Meanwhile, the contents of oligomers obtained from hemicellulose degradation were higher than that of monomers, suggesting that lower severity (log *R*_o_) of 2.06 for 30 min and 2.37 for 60 min at 120 °C favored the production of oligomers and was insufficient for the degradation of the generated oligomers to monomers [21].

### Characterization of the crystallinity, surface morphology, and thermogravimetry of native and pretreated sugarcane bagasse

**Fig. 1:**
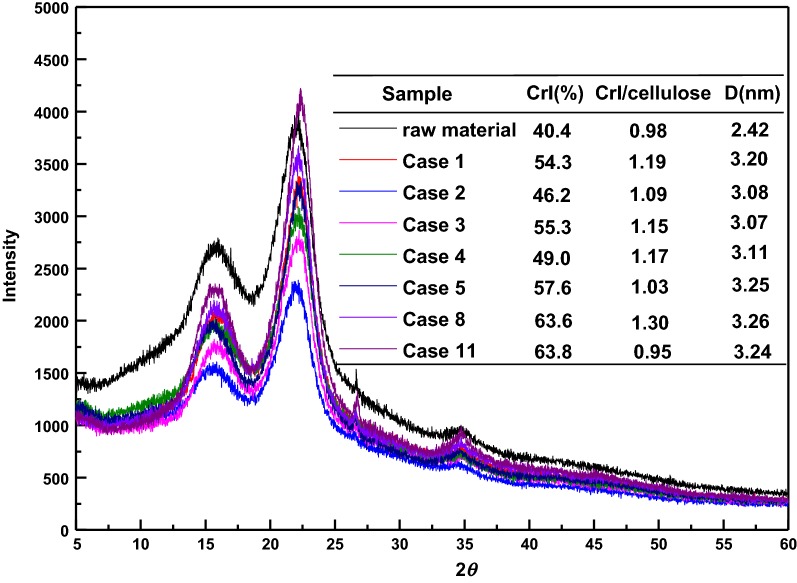
The crystallinity indexes of raw sugarcane bagasse and pretreated substrates

It was reported that pretreatment could transform the lignocellulose crystallinity by opening crystal hydrogen bonding, degrading amorphous constituents, and increasing crystal regions, which would affect the subsequent enzymatic saccharification [22]. Hence, the XRD patterns and CrI of untreated and pretreated substrates were investigated and are represented in Fig. 1. As presented, the CrI of native sugarcane bagasse was 40.4%. After pretreatment, all the pretreated substrates presented higher CrIs than the control (raw material). The CrIs of Case 1, Case 2, and Case 3, which were subjected to alkali alone, ethanol alone, and the combination of alkali and ethanol pretreatment, increased by 34.4%, 14.4%, and 36.9%, respectively. This phenomenon was attributed to the removal of amorphous hemicellulose and lignin, which was consistent with the chemical composition analysis presented in Table 1. It was also found that the increased CrIs presented a positive correlation with the removal of the total amounts of the amorphous portion [23]. Similar results could be observed for substrates pretreated with liquid hot water (Case 4) and dilute acid (Case 5) pretreatment, yielding CrIs of 49% and 57.6% with increased yields of 21.3% and 42.6%, respectively. Considering the pretreated substrates of Case 4 and Case 5 for second-step pretreatment, their CrIs reached 63.6% for Case 8 and 63.8% for Case 11, respectively, which were obviously higher than that of the one-step process substrate. The increased yields of CrIs were 30.4% and 10.8%, respectively, which presented an inverse tendency to that pretreated by the one-step process, suggesting more amorphous portions (including hemicellulose and lignin) were removed during the second-step pretreatment. This has been verified in Table 1. This elimination led to the rupture of the intact structure, exposing more cores and fragments, providing more cellulose for the accessibility of enzyme [24, 25]. However, the ratio of CrI to the cellulose content also presented the same trend with CrI, increasing to 1.03–1.30 by one-step pretreatment or by combinatorial pretreatment except in Case 11, compared to the initial 0.98. This phenomenon was ascribed to the removal of amorphous cellulose during pretreatment (shown in Table 1), which led to the increment of crystalline cellulose [26]. For Case 11, the removal of cellulose reached 10.1% and the ratio of CrI to cellulose decreased to 0.95, suggesting that a certain amount of crystalline cellulose was removed.

Furthermore, cellulose crystallite size (D) of untreated and pretreated substrates has been also calculated as shown in Fig. 1. The crystallite size of raw sugarcane bagasse was 2.28 nm (002). For pretreated substrates, cellulose crystallite size was larger than that of native material, showing similar tendencies of the performance change with CrIs.

When two-step pretreatments were implemented, the crystallite size increased gradually to 3.26 nm (Case 8) and 3.24 nm (Case 11), respectively. This phenomenon indicated that pretreatment did not disrupt the cellulose crystallinity and reduce the crystalline size, but increased it, due to the reformation or recrystallization of crystalline cellulose [27].

**Fig. 2:**
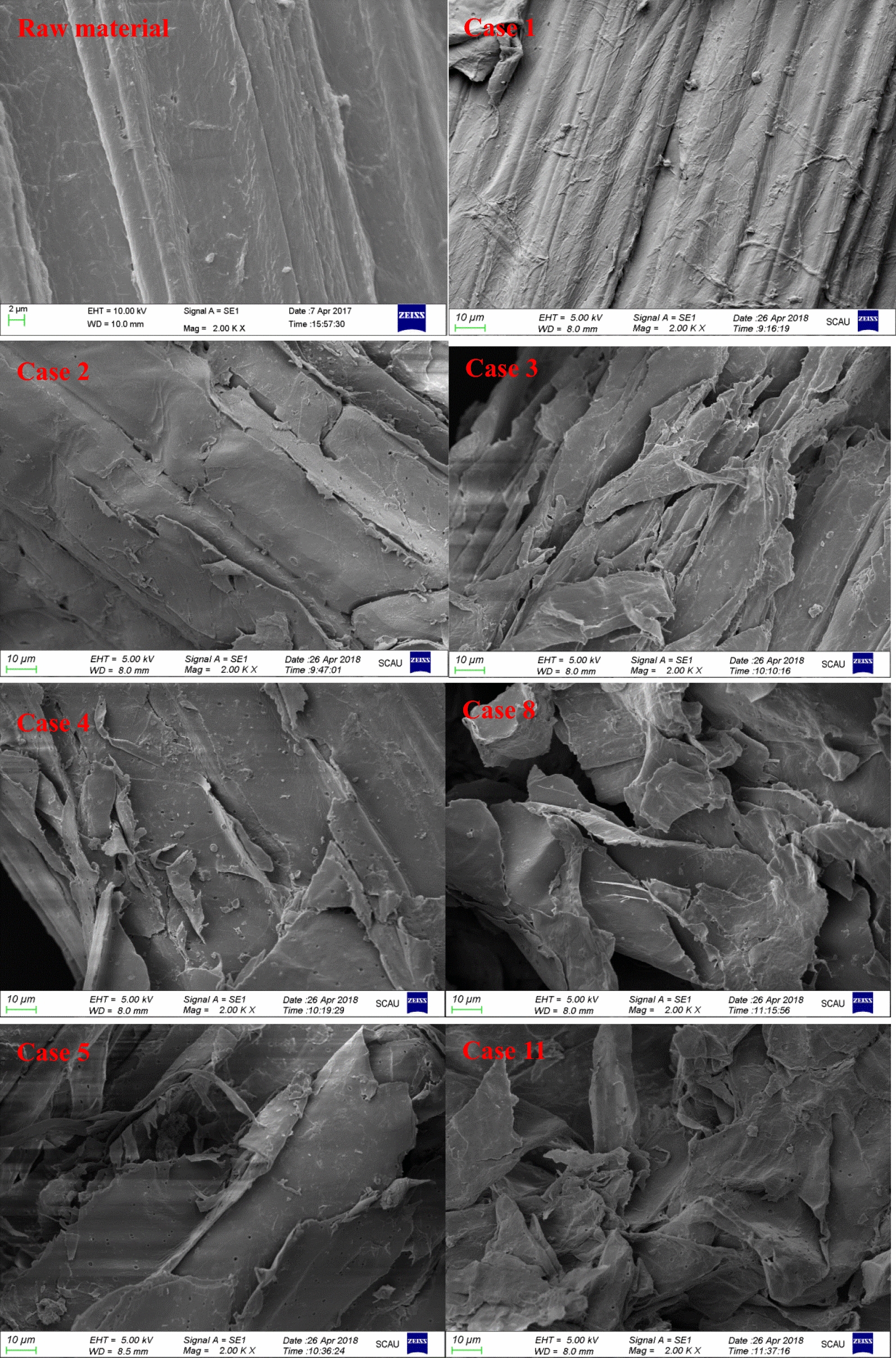
SEM images of raw material and pretreated substrates at ×2000 magnification

The ultrastructure of untreated and pretreated solids has been captured by SEM as shown in Fig. 2. As was shown, the untreated material had a compact and highly fibrillary morphology with an intact and smooth surface, which impeded the accessibility of enzyme to cellulose [3]. The sample treated with 0.5% NaOH in Case 1 presented a similar morphology to that of the native material. When exposed to other one-step pretreatments, the matrix fibrillary structures were broken, became loose, and separated with a lot of fragments and gullies due to the removal of hemicellulose/lignin. This result indicated that the pretreatment disrupted the physically structural barrier of biomass and exposed more cellulose with more surface area and roughness, providing more reactive sites for the enzymatic saccharification [28]. When two-step pretreatment was conducted, obvious collapsed surface with numerous pores, cracks, and lamellar fibers was presented. However, the enzymatic efficiency was not inconsistent with the removal of hemicellulose/lignin. It was possible that the large removal of hemicellulose and lignin led to the collapsed cellulose, which provided less reactive sites for the accessibility of enzyme.

**Fig. 3:**
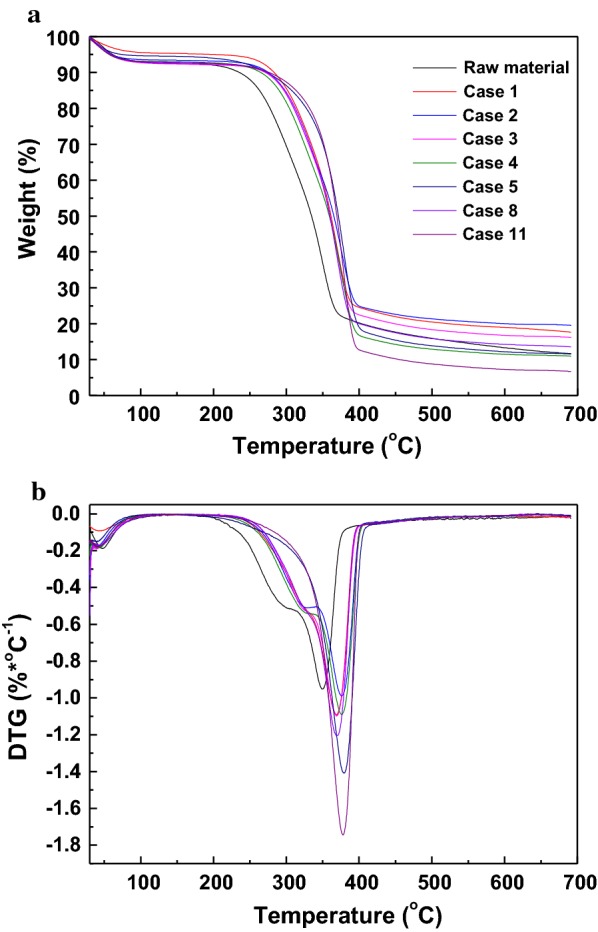
TG and DTG distributions of raw material and pretreated substrates

Thermogravimetric analysis of untreated and pretreated sugarcane bagasse was performed to determine the content change and the thermal stability of the composites, and the mass loss and derivative curves are shown in Fig. 3a, b. The original weight loss below 110 °C for all samples was due to water evaporation. For untreated biomass, there were two decomposition peaks at 304 and 349 °C, attributed to the degradation of hemicellulose and cellulose/lignin. The crystalline structure of cellulose and aromatic/dimensional structure of lignin contributed to the higher degradation temperature than amorphous hemicellulose [29]. For one-step pretreated substrates excepting Case 5 and two-step pretreated substrate of Case 8, two weight loss peaks could be observed, suggesting that most of hemicellulose were reserved in pretreated substrates. This phenomenon indicated that the one-step pretreatments proposed in this study were not enough for the large decomposition of hemicellulose, as confirmed in Table 1. For Case 5 and Case 11, as about 75–85% of hemicellulose was removed, there was only one weight loss peak due to the decomposition of retained cellulose and lignin, which was in agreement with the disappearance of the hemicellulose peak [30]. However, compared to untreated material (335 °C), the thermal decomposition temperatures of pretreated substrates for 50% weight loss were increased to different degrees and reached 361, 365, 360, 358, 374, 356, 361 °C for Case 1–5, Case 8, and Case 11, respectively. This phenomenon indicated that the reduction of hemicellulose and lignin in the pretreated solids led to the increment of the degradation temperatures during the TG process. In addition, the degradation rate of the largest peak increased gradually from − 0.9 to − 1.7%/°C, due to the higher content of cellulose and less content of lignin in the pretreated substrates, which was confirmed by the higher decomposition temperature of lignin (> 360 °C) than cellulose (320–400 °C).

### Combinatorial pretreatment improved cellulose conversion

**Fig. 4:**
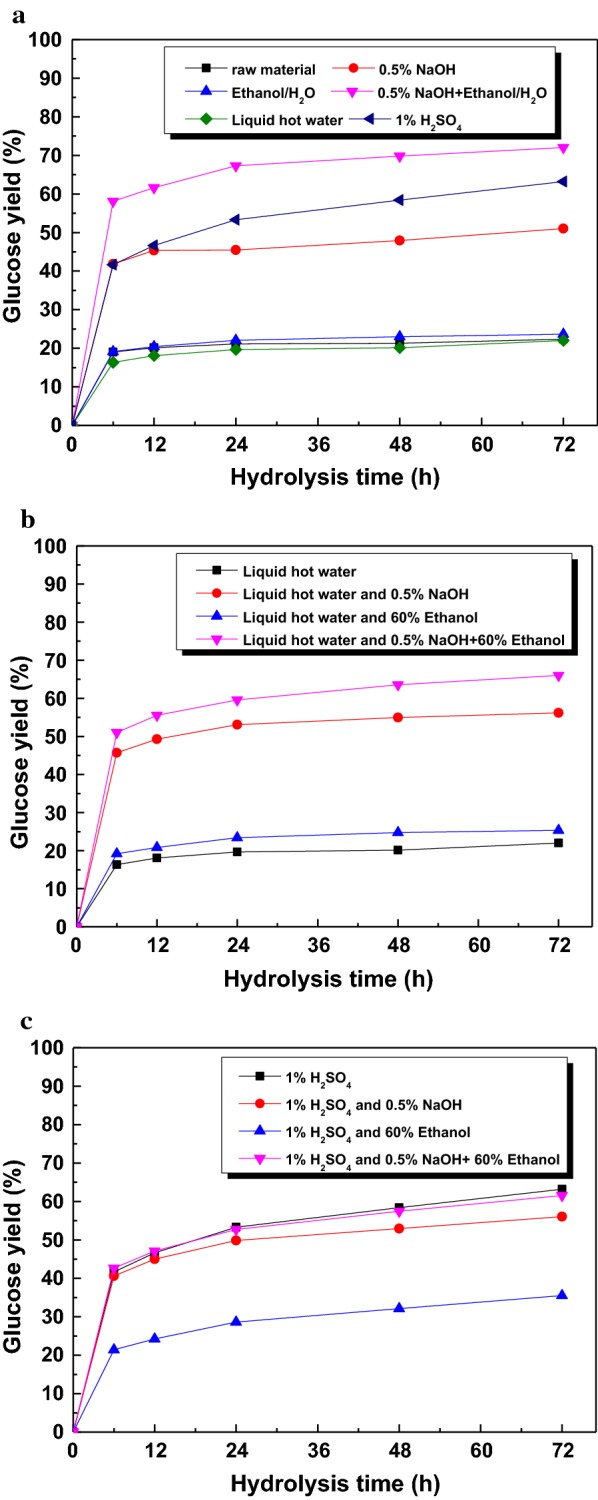
Glucose yield after combinatorial pretreatment with enzyme loading of 20 FPU/g substrate

The enzymatic digestibility of the pretreated substrates was determined and their glucose yields are shown in Fig. 4. Figure 4a illustrated the glucose yield obtained from native material and one-step pretreated substrates with enzyme loading of 20 FPU/g substrate. For the native material, the glucose yield after 72 h only reached 22.4%. When sugarcane bagasse was pretreated with 0.5% NaOH (Case 1) or 60% ethanol alone (Case 2) or the combination of them (Case 3), the final glucose yields (at 72 h) were 51, 23.6, and 72.0%, respectively. This result indicated that at mild conditions (120 °C and 30 min), NaOH pretreatment presented a better performance than 60% ethanol pretreatment on the improvement of glucose yield. The glucose yield of the pretreated solid with the combinatorial pretreatment of NaOH and 60% ethanol (Case 3) was substantially enhanced by 41% and 205% compared to that of NaOH or 60% ethanol pretreated solids, respectively, which was in line with a previous report [1]. This phenomenon was ascribed to the higher removal of hemicellulose (22.6% of xylan) and lignin (17.9% of AIL), which destroyed the matrix structure and provided more cellulose for enzyme accessibility [16, 27].

When liquid hot water was used for sugarcane bagasse pretreatment, there was no improvement in glucose yield (22.0%) (Case 4), suggesting that liquid hot water at 120 °C did not cause acute change in biomass structure, which was in agreement with the slight removal of xylan (3.8%) and AIL (1.5%) shown in Table 1. When the liquid hot water pretreated solids were used as substrates for the second-step pretreatment (Fig. 4b), the glucose yields after 72 h with enzyme loading of 20 FPU/g substrate reached 56.2% (Case 6), 25.3% (Case 7), and 66% (Case 8), respectively. Compared with one-step pretreatment with liquid hot water, the glucose yields presented different degrees of increment after two-step pretreatment. However, when compared to the one-step pretreatment with NaOH or 60% ethanol, the two-step pretreated substrate gave rise to a slight increment in glucose yield. When comparing with the combination of them, a reduction of glucose yield was observed, which was consistent with the lower removal of hemicellulose and higher delignification. This result may understood by the fact that the large removal of lignin (60.5%) induced the collapse of the fiber structure and the cellulose was wrapped by the retained hemicellulose and lignin, which impeded the enzymatic attack and accessibility of the enzyme [31].

When the sugarcane bagasse was pretreated with 1% H_2_SO_4_, the glucose yield after 72 h hydrolysis reached 63.2% (Case 5). When second-step pretreatment was conducted, the glucose yields after enzymatic hydrolysis for 72 h with enzyme loading of 20 FPU/g substrate were 56.1% (Case 9), 35.5% (Case 10), and 61.6% (Case 11), respectively, which were lower than that obtained from the first-step pretreated substrate. This phenomenon was also ascribed to the large removal of hemicellulose (80.6–85.6% of xylan) and lignin (23.1–55.3% of AIL), which destroyed the matrix structure, collapsed the fiber structure, disputed the accessibility of enzyme, and induced the enzymatic efficiency [32]. At the same time, the pseudo-lignin generated during the dilute acid pretreatment was precipitated on the surface, hindering the subsequent enzymatic hydrolysis by blocking the surface binding sites, or contributing to the non-productive binding between lignin and cellulases due to its hydrophobic nature [33]. However, the glucose yields obtained from two-step pretreated substrates with NaOH or 60% ethanol were higher than that one-step pretreated solids with the same chemicals, due to the large removal of hemicellulose and lignin, which destroyed the intact structure and provided more reactive sites for cellulase (as shown in Fig. 2) [34]. When a combinatorial pretreatment of them was used, the glucose yield obtained from the two-step pretreated substrate was lower than that from the one-step pretreated solid, which may be caused by lignin precipitation and modification on the fiber surface, resulting in the hindrance of enzyme attack [19].

### Effect of surfactant addition on carbohydrate conversion

**Fig. 5:**
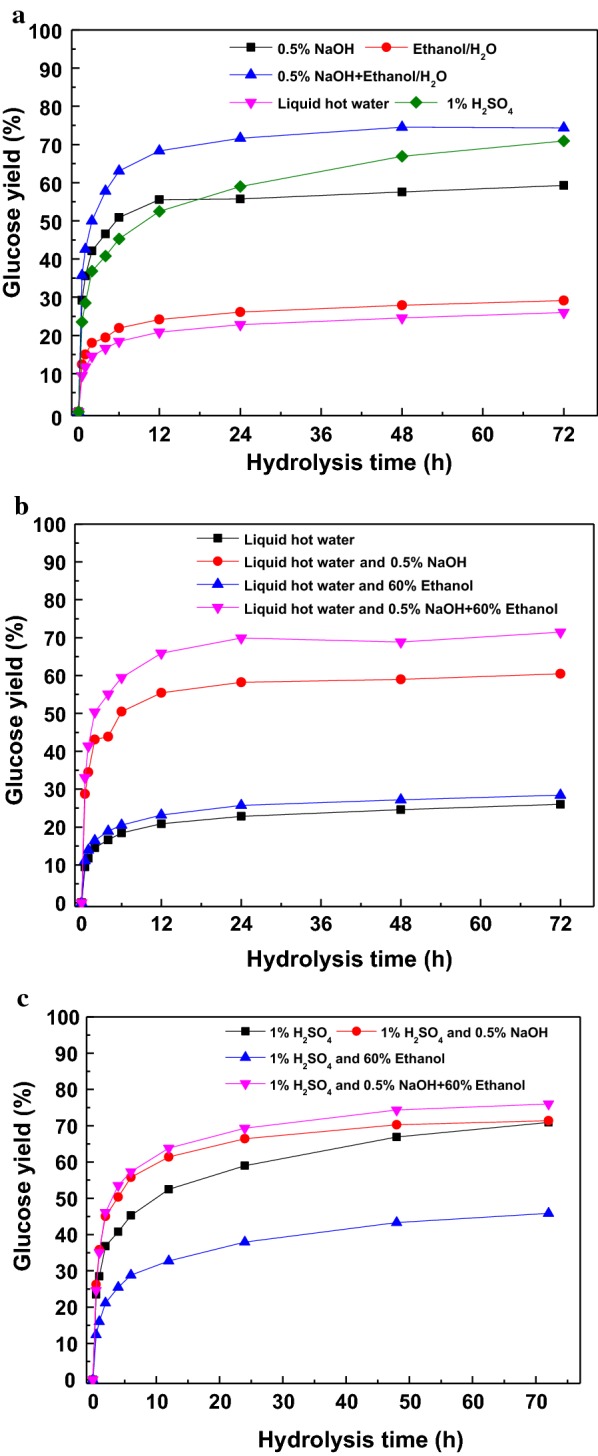
The enhancement of Tween 80 (150 mg/g pretreated substrate) on glucose yield of different pretreated substrates with enzyme loading of 20 FPU/g substrate

As shown in Fig. 4, some of the two-step pretreated substrates did not present distinct improvement in glucose yield compared with that pretreated with one-step pretreatment. Hence, it is necessary to explore effective methods to improve enzymatic hydrolysis. The influence of Tween 80 on the enzymatic digestibility of various pretreated substrates was determined and the glucose yields are illustrated in Fig. 5 and Additional file 1: Figure S1. Compared with that shown in Fig. 4, the glucose yields increased gradually with the addition of Tween 80. For one-step pretreatment, the glucose yields after 72 h were 59.3%, 29.1%, 74.3%, 26.0%, and 70.9% for Case 1–5, respectively, with increased glucose yields of 16.2%, 23.2%, 3.2%, 18.2%, and 12.3%. This improvement was ascribed to lubricating the access of cellulase to cellulose and reducing the non-productive adsorption of cellulase to lignin with the addition of Tween 80 [9, 14]. Though the substrate pretreated with a combination of NaOH and 60% ethanol generated the maximum glucose yield of 74.3%, the minimum increased glucose yield of 3.2% suggested that the combinatorial pretreatment did play a leading role in improving the enzymatic hydrolysis, which was in accordance with our reports that the improvement of Tween 80 became weak at relative high glucose yields due to the lack of cellulose-rich substrate [35].

When liquid hot water pretreated substrates were used for second-step pretreatment (Fig. 5b), the combinatorial pretreated substrate with NaOH and 60% ethanol presented a comparatively good glucose yield of 71.4% (Case 8), followed by Case 6 (60.4%) and Case 7 (28.4%), with increased glucose yields of 8.2%, 7.5%, and 12.4%, respectively, which were lower than that with one-step pretreatment. This result is attributed to the low content of lignin in the pretreated substrates, which provided limited enhancement in enzymatic hydrolysis [17]. As shown in Fig. 5c, the synergy of combination of 1% H_2_SO_4_ pretreatment with various second-step pretreatments did increase the glucose yields to 71.4% (Case 9), 45.9% (Case 10), and 76% (Case 11) with increased glucose yields of 27.3%, 29.1%, and 23.4%, respectively. The highest glucose yield (76%) was obtained with two-step pretreatment (Case 11) and the addition of Tween 80, liberating 84.5% of theoretical glucose in the pretreated substrate. The higher increased glucose yield was ascribed to the large removal of hemicellulose, which provided higher accessibility to Tween 80 binding with lignin, decreasing the non-productive enzymes, providing more enzyme for enzymatic digestibility, and augmenting the glucose yield [36].

**Fig. 6:**
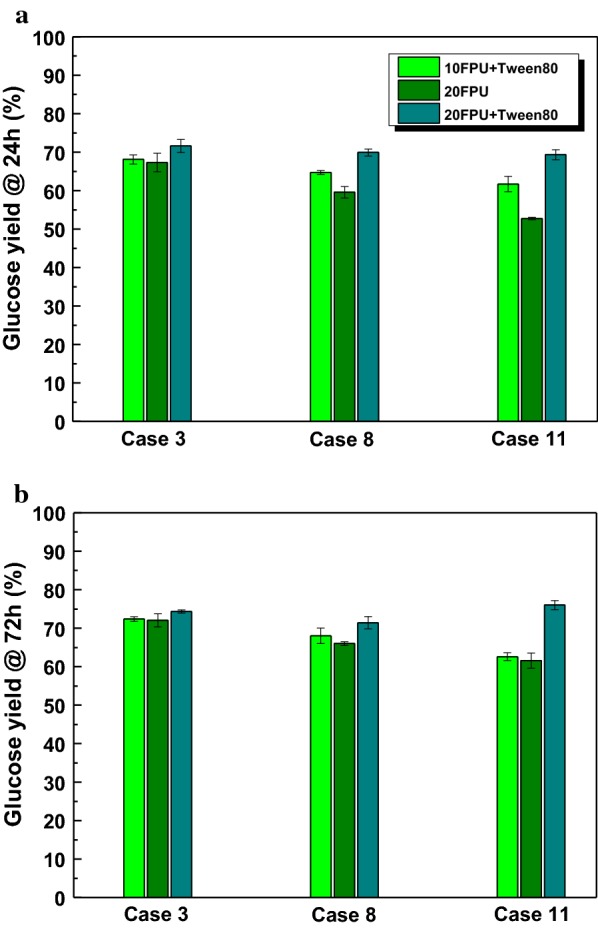
Glucose yields obtained from three different pretreated substrates with the addition of Tween 80 (150 mg/g pretreated substrate) under hydrolysis time of 24 h (**a**) and 72 h (**b**)

As shown in Figs. 4 and 5, the addition of Tween 80 at relatively high loading of enzyme (20 FPU/g substrate) could gradually improve the enzymatic hydrolysis and enhance the glucose yield. When half of the enzyme loading was reduced, it is necessary to investigate how Tween 80 enhanced the enzymatic hydrolysis; the liberated glucose yields from Case 3, Case 8, and Case 11 after hydrolysis for 24 h and 72 h are shown in Fig. 6a, b, respectively. As depicted in Fig. 6a (24 h), though the glucose yields obtained with 20 FPU enzyme and Tween 80 generated the highest glucose yields for three different pretreated substrates, the glucose yields gained with 10 FPU enzyme and Tween 80 were 68.1%, 64.7%, and 61.7%, respectively, which were a little higher than that with the 20 FPU enzyme without Tween 80, suggesting that the addition of Tween 80 could save more than 50% of enzyme [35]. The addition of Tween 80 to 20 FPU/g substrate led to increased glucose yields of 6.4%, 17.4%, and 31.5% for Case 3, Case 8, and Case 11, respectively. However, it was also found that the glucose yields generated at 20 FPU after 24 h with Tween 80 were similar with that obtained at 20 FPU after 72 h without Tween 80, suggesting that Tween 80 could shorten the hydrolysis time to 24 h at 20 FPU while retaining the same glucose yield. This phenomenon indicated that this condition could reduce two-thirds of the hydrolysis time and save a large quantity of energy during enzymatic hydrolysis, which was in accordance with the reports by Monschein et al. on the addition of PEG 8000 to thermo-acidically pretreated wheat straw [12].

As the hydrolysis time was extended to 72 h (shown in Fig. 6b), with enzyme loading of 10 FPU/g substrate, the glucose yields with Tween 80 reached 72.4%, 68.0%, and 62.6% for Case 3, Case 8, Case 11, respectively. Though this condition also presented slightly higher glucose yields than that obtained with 20 FPU enzyme without Tween 80, the difference among glucose yields became weak as the hydrolysis time was extended from 24 h to 72 h. With the addition of Tween 80 at loading of 20 FPU/g substrate, the glucose yields increased at 3.2%, 8.2%, and 23.4% for Case 3, Case 8, and Case 11, respectively, which were lower than that at 24 h, suggesting that the improvement with Tween 80 became slack as hydrolysis time was extended from 24 h to 72 h, due to the reduction of cellulose and enzyme after holding for a long time [10, 24, 37].

## Methods

### Materials

The sugarcane bagasse used in this study was collected from Shaoguan, China. It was ground to a powder (~ 1 mm) using a mill and stored in sealed bag for further investigation. The chemical constituents of native material were determined according to the National Renewable Energy Laboratory (NREL) protocol and are presented in Table 1 [38].

### Combinatorial pretreatment strategies of sugarcane bagasse

Eleven pretreatment strategies were proposed, alone or in combination: sodium hydroxide, ethanol, liquid hot water, and dilute sulfuric acid pretreatment [1]. The pretreatment temperature and time of each strategy are also presented in Table 2. All pretreatments were conducted in a 100 mL screw bottle at 120 °C for 30–60 min by using a non-pressure vessel (steam sterilizer). For single pretreatments (Cases 1, 2, 3, 4, and 5), a certain amount of sugarcane bagasse was loaded into the screw bottle with solid/liquid ratio of 1:10. After the reaction completed, the pretreated slurry was separated by filtration, washed with deionized water several times, and stored in a refrigerator for further investigation. The liquids were collected for sugar analysis. Oligomers were calculated based on the increased yields of monomer sugars after autoclaving at 121 °C for 60 min with 4% sulfuric acid [38]. For combinatorial pretreatments (Cases 6, 7, 8, 9, 10, and 11), sugarcane bagasse (loaded as described above) was pretreated with liquid hot water or dilute H_2_SO_4_ in step one, followed by 0.5% NaOH solution, or 60% ethanol solution, or the combination of them in step two.

**Table 2:** Combinatorial pretreatment strategies of sugarcane bagasse at 10% (w/w) loading

Case	Step 1		Step 2	
	Chemicals	Conditions	Chemicals	Conditions
1	0.5% NaOH	120 °C, 60 min		
2	60% ethanol	120 °C, 60 min		
3	60% ethanol + 0.5% NaOH	120 °C,60 min		
4	Liquid hot water	120 °C, 30 min		
5	1% H_2_SO_4_	120 °C, 30 min		
6	Liquid hot water	120 °C, 30 min	0.5% NaOH	120 °C,60 min
7	Liquid hot water	120 °C, 30 min	60% ethanol	120 °C, 60 min
8	Liquid hot water	120 °C, 30 min	60% ethanol + 0.5%NaOH	120 °C, 60 min
9	1% H_2_SO_4_	120 °C, 30 min	0.5% NaOH	120 °C, 60 min
10	1% H_2_SO_4_	120 °C, 30 min	60% ethanol	120 °C, 60 min
11	1% H_2_SO_4_	120 °C, 30 min	60% ethanol + 0.5%NaOH	120 °C, 60 min

### Enzymatic hydrolysis of sugarcane bagasse

The untreated and pretreated sugarcane bagasse was hydrolyzed using Cellic CTec2 (90 FPU/mL) with an enzyme loading of 20 FPU/g dry pretreated substrate [39]. Enzymatic hydrolysis assays were managed in 50 mM sodium acetate buffer solution (pH 4.8) at 2% (w/v) solid loading in a shaker at 50 °C and 150 rpm [35]. After hydrolysis for 6, 12, 24, 48, and 72 h, a small amount of supernatant was collected for analysis.

To determine the influence of Tween 80 on the enzymatic digestibility, it was added into mixture at a loading of 150 mg/g dry pretreated substrate and incubated for 30 min for complete interaction between substrate and additive before the addition of enzyme [35]. The recording of hydrolysis time was started when enzyme was added into the mixture.

### Analytical methods

The chemical composition of untreated and treated material, pretreatment liquors, and glucose yields were detected by the HPLC system (Shimadzu, Japan) equipped with a cation-exchange column of SUGAR SH1011 and a refractive index (RI) detector at 50 °C with 1.0 mL/min of H_2_SO_4_ at 5 mM as eluates. The glucose yields obtained from enzymatic digestibility and the increased yields of glucose with Tween 80 were calculated based on the following equations:

$${\text{Glucose}}\;{\text{Yield }}( \, \% ) = \frac{{{\text{glucose}}\;{\text{produced}}\;{\text{in}}\;{\text{enzymatic}}\;{\text{hydrolysis}}}}{{{\text{glucan}}\;{\text{amount}}\;{\text{in}}\;{\text{raw}}\;{\text{ material }}* \, 1.11}} \times 100\% ,$$

$${\text{Increased}}\;{\text{Yield }}( \, \% ) = \frac{{{\text{glucose}}\;{\text{yield}}\;{\text{with}}\;{\text{surfactant}} - {\text{glucose}}\;{\text{yield}}\;{\text{without}}\;{\text{surfactant}}}}{{{\text{glucose}}\;{\text{yield}}\;{\text{without}}\;{\text{surfactant}}}} \times 100\% .$$

### Characterization of untreated and pretreated sugarcane bagasse

X-ray diffraction patterns of untreated and pretreated sugarcane bagasse were recorded with a Bruker D8-ADVANCE (Karlsruhe, Germany) with Cu radiation (1.542 Å) in the range from 5° to 60°. The crystalline index (CrI) and cellulose crystallites size (D) were calculated based on the Segal method [40] and Scherrer equation [41], respectively. SEM images (×2000) of untreated and pretreated sugarcane bagasse were obtained using an EVO18 (ZEISS, Germany). TG analyses of untreated and pretreated samples were conducted using TG-Q500 (TA instruments, USA). About 5–8 mg samples were heated from room temperature to 700 °C at a rate of 1 °C/min in a nitrogen environment (60 mL/min).

## Conclusions

Combinatorial pretreatments with liquid hot water/H_2_SO_4_ and ethanol/NaOH of sugarcane bagasse were developed to enhance the enzymatic hydrolysis under mild conditions. After one-step 60% ethanol containing 0.5% NaOH pretreatment, the glucose yield was enhanced by 41% and 205% compared to that pretreated with only 0.5% NaOH or 60% ethanol. Multiple detailed physical and chemical characterizations of untreated and pretreated substrates elaborated the mechanism of glucose yield improvement. However, using combinatorial pretreatments with 1% H_2_SO_4_ followed by 60% ethanol containing 0.5% NaOH, the highest glucose yield with Tween 80 reached 76%, representing 84.5% of the theoretical glucose in the pretreated substrate. While retaining similar glucose yield, the addition of Tween 80 enabled either a reduction of enzyme loading by 50% or shortening of hydrolysis time to 24 h. However, the enhancement of Tween 80 decreased as the hydrolysis time was extended. The present study demonstrated that the two-step process based on successive H_2_SO_4_ and ethanol/NaOH treatment with the addition of Tween 80 provided a promising technology to achieve high glucose yield from sugarcane bagasse.

## Additional file

**Additional file 1: Table S1.** Components of pretreatment liquor with each pretreatment. **Figure S1.** The glucose yields obtained after 72 h and increases yields (shown on the top of column) with addition of Tween 80 (150 mg/ g substrate).
