# Supplementary material for: Enhancing enzymatic saccharification of sugarcane bagasse by combinatorial pretreatment and Tween 80
Source: Biotechnol Biofuels. 2018 Nov 9;11:309. doi: 10.1186/s13068-018-1313-7 (PMC6225707; doi:10.1186/s13068-018-1313-7)
Supplement: Supplementary file 1 — Additional file 1: Table S1. Components of pretreatment liquor with each pretreatment. Figure S1. The glucose yields obtained after 72 h and increases yields (shown on the top of column) with addition of Tween 80 (150 mg/ g substrate). [file 13068_2018_1313_MOESM1_ESM.docx]

**Enhancing enzymatic saccharification of sugarcane bagasse by combinatorial pretreatment and Tween 80**

**Hongdan Zhang^a,b,c*^, Weiqi Wei^c^, Jiajie Zhang^a^, Shihang Huang****^a^, Jun Xie^a^**

^a^ College of Forestry and Landscape Architecture, Key Laboratory of Energy Plants Resource and Utilization, Ministry of Agriculture, South China Agricultural University, Guangzhou 510642, P.R. China

^b^ State Key Laboratory of Pulp and Paper Engineering, South China University of Technology, Guangzhou 510640, P.R. China

^c^ College of Light Industry and Food Engineering, Nanjing Forestry University, Nanjing 210037, P.R. China

Corresponding author: Hongdan Zhang; E-mail address: [hdzhang@scau.edu.cn](mailto:hdzhang@scau.edu.cn).

**Table S1** Components of pretreatment liquor with each pretreatment.

| Pretreat  -ment | Sugar analysis (g/100g raw material) | | | | | | | | | | | |
| --- | --- | --- | --- | --- | --- | --- | --- | --- | --- | --- | --- | --- |
|  | Arab.  Mono. | Arab.  Olig. | Total  Arab. | Gala.  Mono. | Gala.  Olig. | Total  Gala. | Xyl.  Mono. | Xyl.  Olig. | Total  Xyl. | Glu.  Mono. | Glu.  Olig. | Total  Glu. |
| Case 1 | 0.00 | 0.82 | 0.82 | 0.00 | 0.49 | 0.49 | 0.00 | 0.92 | 0.92 | 0.00 | 0.54 | 0.54 |
| Case 2 | 0.03 | 1.11 | 1.14 | 0.02 | 0.66 | 0.68 | 0.00 | 2.03 | 2.03 | 0.02 | 0.74 | 0.76 |
| Case 3 | 0.01 | 1.26 | 1.27 | 0.00 | 0.75 | 0.75 | 0.00 | 2.67 | 2.67 | 0.00 | 0.86 | 0.86 |
| Case 4 | 0.00 | 0.26 | 0.26 | 0.00 | 0.15 | 0.15 | 0.00 | 0.24 | 0.24 | 0.00 | 0.39 | 0.39 |
| Case 5 | 0.59 | 0.78 | 1.38 | 0.48 | 0.34 | 0.82 | 1.49 | 1.65 | 3.14 | 0.44 | 0.47 | 0.91 |
| Case 6 | 0.01 | 0.59 | 0.60 | 0.01 | 0.35 | 0.35 | 0.01 | 1.11 | 1.11 | 0.00 | 0.13 | 0.13 |
| Case 7 | 0.03 | 0.09 | 0.12 | 0.02 | 0.05 | 0.07 | 0.00 | 0.13 | 0.13 | 0.00 | 0.04 | 0.05 |
| Case 8 | 0.00 | 0.14 | 0.14 | 0.00 | 0.08 | 0.08 | 0.00 | 0.19 | 0.19 | 0.00 | 0.08 | 0.09 |
| Case 9 | 0.01 | 0.09 | 0.10 | 0.01 | 0.05 | 0.06 | 0.00 | 0.46 | 0.47 | 0.00 | 0.08 | 0.08 |
| Case10 | 0.01 | 0.12 | 0.13 | 0.01 | 0.07 | 0.08 | 0.01 | 0.19 | 0.20 | 0.00 | 0.02 | 0.03 |
| Case11 | 0.01 | 0.10 | 0.11 | 0.01 | 0.06 | 0.07 | 0.01 | 0.33 | 0.34 | 0.00 | 0.08 | 0.08 |


**Figure S1** The glucose yields obtained after 72 h and increases yields (shown on the top of column) with addition of Tween 80 (150 mg/ g substrate).
